# Prevalence of Cataract in an Older Population in India: The India Study of Age-related Eye Disease

**DOI:** 10.1016/j.ophtha.2010.05.020

**Published:** 2011-02

**Authors:** Praveen Vashist, Badrinath Talwar, Madhurjya Gogoi, Giovanni Maraini, Monica Camparini, Ravilla D. Ravindran, Gudlavalleti V. Murthy, Kathryn E. Fitzpatrick, Neena John, Usha Chakravarthy, Thulasiraj D. Ravilla, Astrid E. Fletcher

**Affiliations:** 1 Dr. Rajendra Prasad Centre for Ophthalmic Sciences, All India Institute of Medical Sciences, New Delhi, India; 2 Aravind Eye Hospital Pondicherry, Aravind Eye Care, Pondicherry, India; 3 Dipartimento di Scienze Otorino-Odonto-Oftalmologiche e Cervico Facciali, Sezione di Oftalmologia, Universita‘ degli Studi di Parma, Parma, Italy; 4 Epidemiology and Population Health, London School of Hygiene & Tropical Medicine, London, UK; 5 Ophthalmology and Vision Science, Queen's University Belfast, Belfast, UK; 6 Lions Aravind Institute of Community Ophthalmology, Madurai, India

## Abstract

**Purpose:**

To describe the prevalence of cataract in older people in 2 areas of north and south India.

**Design:**

Population-based, cross-sectional study.

**Participants:**

Randomly sampled villages were enumerated to identify people aged ≥60 years. Of 7518 enumerated people, 78% participated in a hospital-based ophthalmic examination.

**Methods:**

The examination included visual acuity measurement, dilatation, and anterior and posterior segment examination. Digital images of the lens were taken and graded by type and severity of opacity using the Lens Opacity Classification System III (LOCS III).

**Main Outcome Measures:**

Age- and gender-standardized prevalence of cataract and 95% confidence intervals (CIs). We defined type of cataract based on the LOCS III grade in the worse eye of: ≥4 for nuclear cataract, ≥3 for cortical cataract, and ≥2 for posterior subcapsular cataract (PSC). Any unoperated cataract was based on these criteria or ungradable dense opacities. Any cataract was defined as any unoperated or operated cataract.

**Results:**

The prevalence of unoperated cataract in people aged ≥60 was 58% in north India (95% CI, 56–60) and 53% (95% CI, 51–55) in south India (*P* = 0.01). Nuclear cataract was the most common type: 48% (95% CI, 46–50) in north India and 38% (95% CI, 37–40) in south India (*P*<0.0001); corresponding figures for PSC were 21% (95% CI, 20–23) and 17% (95% CI, 16–19; *P* = 0.003), respectively, and for cortical cataract 7.6% (95% CI, 7–9) and 10.2% (95% CI, 9–11; *P*<0.004). Bilateral aphakia/pseudophakia was slightly higher in the south (15.5%) than in the north (13.2%; *P*<0.03). The prevalence of any cataracts was similar in north (73.8%) and south India (71.8%). The prevalence of unoperated cataract increased with age and was higher in women than men (odds ratio [OR], 1.8). Aphakia/pseudophakia was also more common in women, either unilateral (OR, 1.2; *P*<0.02) or bilateral (OR, 1.3; *P*<0.002).

**Conclusions:**

We found high rates of unoperated cataract in older people in north and south India. Posterior subcapsular cataract was more common than in western studies. Women had higher rates of cataract, which was not explained by differential access to surgery.

**Financial Disclosure(s):**

The authors have no proprietary or commercial interest in any of the materials discussed in this article.

Cataract is a major cause of vision impairment in many low-income settings.^1^ It remains uncertain as to whether the high levels observed are explained largely by reduced access to cataract surgery or additionally to potential environmental risk factors more prevalent in low-income settings, such as poor diets, occupational sunlight exposure, and use of biomass fuels. Genetic factors may also be relevant, especially if cataract prevalence varies between low-income populations. Variations in the prevalence of different types of cataract may also suggest possible etiologic or genetic factors. The evidence to date using comparable methods of cataract measurement that include untreated opacities and aphakia/pseudophakia generally supports a higher prevalence of cataract in various Asian populations^2^ compared with Western populations.^3^ India is a vast country with substantial geographical variation, for example, in climate, dietary patterns, and ancestry, but there have been very few prevalence studies (only 2 previous studies in the south and a small feasibility study for the present study in the north).^4–6^ We undertook a 2-center study in north and south India using a common protocol to examine the prevalence and risk factors for cataract. The present paper reports the results for cataract prevalence.

## Methods

The India Study of Age-related Eye Disease (INDEYE study) is a population-based study of people aged ≥60 years. The objectives of the INDEYE study were to estimate the age and gender specific prevalence of early and late age-related macular degeneration and of lens opacities, and to investigate associations with these conditions and tobacco use, exposure to biomass cooking fuels, outdoor work, and dietary factors. In this paper, we report the results for the prevalence of cataract.

The study took place in 2 locations: Gurgaon district, in Haryana state, north India, and Pondicherry and Cuddalore district in Tamil Nadu, south India. These areas include rural and urban (small towns) populations served by the participating eye hospitals (Dr Rajendra Prasad Centre for Ophthalmic Sciences [RPC], the All India Institute of Medical Sciences, Delhi, and the Aravind Eye Hospital [AEH], Pondicherry). Gurgaon city and Pondicherry city were excluded because of the high mobility in and out of these 2 locations and mixed ethnicity. A total of 59 clusters—29 in north India and 30 in south India—were randomly selected on the basis that 8% of the total population would be aged ≥60 years. The study aimed to enroll 3000 people aged ≥60 years in each of the 2 study centers allowing for a response rate of around 80%. The sample size calculations were based on the estimated prevalence of age-related macular degeneration from an earlier feasibility study.^7^ With these numbers, we had high power to estimate the prevalence of cataract because cataract is much more common than age-related macular degeneration.

Before the start of the study enumeration, meetings were held with local village leaders to explain the study objectives and methods. A total of 7518 people aged ≥60 years—3586 in north India and 3932 in south India—were identified from enumeration and invited to take part in the study. Recruitment into the study was carried out between 2005 and 2007. Participants who were illiterate had the information leaflet read to them and subjects were enrolled into the study only after informed written consent (for illiterate participants, this consisted of a thumb impression) was obtained. The study complied with the guidelines in the Declaration of Helsinki and ethics approval was received from the Research Ethics Committees of the All India Institute of Medical Sciences, Aravind Eye Hospital, London School of Hygiene and Tropical Medicine, and Queen's University Belfast.

### Study Procedures

Household sociodemographic data were collected at enumeration. Participants were interviewed at home by trained fieldworkers using a structured questionnaire, which included tobacco and alcohol use, cooking fuels and practices, and outdoor work. Diet was assessed by 24-hour recall. Within 1 week of the home interview, participants were brought to the base hospital for the clinical examination, which included anthropometry, blood pressure, an eye examination, and blood sample collection. In the case of refusals of the clinical examination, participants were recontacted at least once and up to 3 times for people who were unavailable.

### Eye Examination

Visual acuity (VA) was tested in each eye separately with the subject wearing habitual spectacles (if any) using the tumbling E Early Treatment of Diabetic Retinopathy Study chart and recorded as Snellen equivalent (≥4 of 5 letters correctly identified in each row). If VA in either of the 2 eyes of a participant was worse than logarithm of the minimum angle of resolution 0.6, refraction was performed using a Nikon (Tokyo, Japan) autorefractor and best-corrected acuity was recorded. Pupillary dilation to ≥6 mm was achieved using 1% tropicamide after anterior segment biomicroscopy. A clinical examination of each eye was performed, which included anterior and posterior segment assessments using slit-lamp biomicroscopy. Fundus photography was undertaken using the Topcon TRC 50 EX fundus acquisition system with preinstalled IMAGEnet software and high-resolution Nikon camera. Digital slit-beam images of the lens were taken using the Topcon SL-D7 Digital photo slit lamp for nuclear opacities (Topcon, Tokyo, Japan) with a resolution of 2048 × 1536 pixels. Retroillumination images of the lens were taken using the Neitz CT-S Cataract Screener for cortical and posterior subcapsular cataract opacities (PSC; Neitz Instruments Ltd., Tokyo, Japan) with a resolution of 640×480 pixels. Before starting the study, the Topcon slit-lamp beam was modified to ensure comparability with other eye surveys using this method of lens photography. The slit width and height were fixed at 0.2 and 9.0 mm, respectively, and the slit beam was locked at 45° at the photographer's left. Two retroillumination lens images (1 focused on the anterior and 1 focused on the posterior lens capsule) were taken on each phakic eye.

### Grading of Lens Images

Lens opacities were graded according to the Lens Opacities Classification System III (LOCS III).^8^ Grading was performed by side-by-side comparison with LOCS III standards placed on a uniformly illuminated background. To grade lens opacities, digital retroillumination images were displayed on a computer screen and adjusted in size to that of the LOCS III standards to facilitate comparison. In both centers, the computer screens used for lens graders had the same illumination and contrast settings. Resolution was set at 1024×768 pixels. No digital enhancement methods were used. Graders assigned a decimal grade in 0.1-unit steps for each opacity up to a maximum of 6.9 for nuclear opacities, and 5.9 for cortical and PSC.

### Quality Assurance

Both photographers and graders at each center were trained by experts at the University of Parma (GM and MC) and certified upon reaching predetermined criteria. Each photographer was certified if all Neitz and Topcon images from 10 eyes were of good quality. Each grader was asked to grade a set of images selected by the University of Parma (20 eyes each for slit lamp Topcon and a different set of pairs of Neitz images from 20 eyes). The criteria for certification were: intraclass correlation coefficient (ICC) with University of Parma graders of ≥0.80; all grades within 2 standard deviations of the mean difference between the grader and Parma gold standard. In total, 7 photographers were certified (4 at the RPC and 3 at the AEH) and 6 graders (4 at the RPC and 2 at AEH). During the study, quality assurance checks were carried out by each grader sending digital images stored on CD to the University of Parma. Every 3 months in the first 6-month period and thereafter every 6 months, images from the last 20 phakic eyes for each photographer were sent to the University of Parma for monitoring of photographic quality. Grading quality was assessed throughout the study from randomly selected images of 20 eyes every 3 months for each grader. These images were independently graded by the University of Parma and quality assurance reports (ICC) generated for each grader. In addition, a random set of digital images from 40 study eyes was exchanged between centers for a 3-way independent grading comparison.

Of the lens photographs from both study centers reviewed during the study, 97% were judged to be of good or fair quality by both study photographers and at the University of Parma (GM and MC). Masked replicate grading of slit-lamp and retroillumination images from 386 eyes from both centers and Parma graders showed an ICC >0.8 for cortical opacities, ≥0.8 for PSC opacities, and >0.9 for nuclear opacities. Similar results were obtained in a cross-check masked replicate grading between RPC and AEH graders on a smaller subset of lens images. The ICCs were 0.9 for all 3 types of opacity.

### Statistical Analysis

Statistical analysis was carried out using Stata 10 (StataCorp, College Station, TX). We defined the type of cataract based on the grade in the worse eye on the LOCS III grade of ≥4 for nuclear cataract, ≥3 for cortical cataract, and ≥2 for PSC. People with any type of cataract based on these criteria or those whose images could not be graded for type of cataract because of dense opacities were included in the definition of any unoperated cataract. People with any unoperated cataract plus those who were pseudophakic or aphakic in either eye were included in the definition of any cataract (i.e., operated plus unoperated cataract). We carried out age and gender standardization using the study population as the standard (direct standardization) to estimate the prevalence of cataract and type of cataract by center. In these analyses, the denominator was all those who underwent the clinical examination. We used logistic regression to investigate the association of age and gender on the prevalence of type of cataract, unoperated cataract, or any cataract. In these analyses the comparator group were those with no cataract or operated cataract (i.e., <4 for nuclear cataract, <3 for cortical cataract, and <2 for posterior subcapsular cataract; no dense opacities and no aphakia/pseudophakia). All analyses took into account the sampling design in the estimation of robust standard errors and corresponding *P*-values and 95% confidence intervals using the ‘survey’ functions in Stata for calculations of rates and design-adjusted Wald tests of significance reported for logistic regression models

## Results

**Figure 1:**
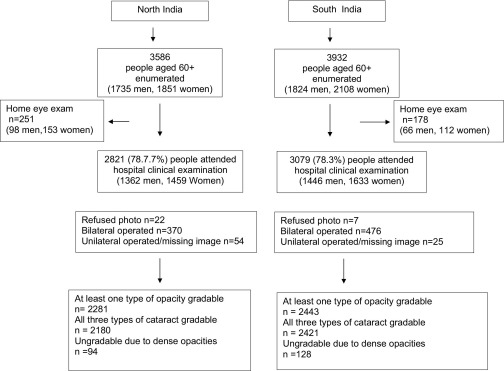
Study flow chart by center.

A total of 7518 people (3932 in north India and 3586 in south India) aged ≥60 years were identified from enumeration (Figure 1). Of those, 429 (5.7%) were examined at home (VA and clinical eye examination) and 5900 (78.5%) attended hospital (AEH or RPC) for an eye examination; 29 refused photography or could not be dilated. Nonresponse to the clinical examination was higher in the oldest age groups, 68% of those aged ≥75 years old underwent the lens examination compared with 80% of those 60 to 74 years old (Table 1; available online at http://aaojournal.org). Otherwise, the differences between responders and nonresponders were small. People with bilateral aphakia/pseudophakia (n = 846) did not undergo lens grading and a further 79 people had aphakia/pseudophakia in 1 eye, but the images from the fellow eye were unavailable. Lens images were available in 4946 people, of which 222 (4.5%) could not be graded owing to bilateral dense lens opacities. Of the remaining 4724 with a LOCS III grade in ≥1 eye, 4555 were gradable for nuclear opacity, 4554 for cortical opacity, and 4552 for PSC opacity; 4487 (76.1%) were gradable for all 3 types of opacity.

Nuclear cataracts were the most common type of opacity and present in 53% of people with a gradable image. These were either pure nuclear (33%) or mixed (20%). Cortical opacities were much less common (11%). In nearly one quarter, PSC was found, mainly mixed PSC and nuclear (15%). There were significant differences between the centers in the age- and gender-standardized prevalence rates of different types of (Table 2). Both pure and mixed nuclear and PSC were more common in the north compared with the south, whereas the prevalence of cortical cataracts was higher in the south.

Over one half (n = 3241) had an unoperated cataract in ≥1 eye with the proportion being slightly higher in the north than in the south (58% and 53%, respectively; *P*<0.01; Table 2). When operated cataracts were included in the definition of a cataract, there were no differences between the centers (74% in the north and 72% in the south) in the age- and gender-standardized prevalence of cataract.

In both centers, the prevalence of cataract (by type or unoperated cataract or unoperated plus operated) increased with age and was higher in women than in men (Table 3; available online at http://aaojournal.org). In analyses comparing those with unoperated cataract with those with no cataract, very high odds ratios (ORs) were observed for the age group aged ≥70 years (OR, 7.01; 95% confidence interval [CI], 5.65–8.70) with ORs compared with those aged 60 to 64 years (Table 4). For combined operated and unoperated cataract the age adjusted OR for women was 1.80 (95% CI, 1.59–2.02).

Nearly one third of people had aphakia/pseudophakia in ≥1 eye; 17% (n = 1014) were unilateral and 14% (n = 846) bilateral. The prevalence of bilateral aphakia was slightly higher in the south (15.5%; 95% CI, 13.6–17.4) compared with the north (13.2%; 95% CI, 11.7–14.7; *P*<0.03), but there was no difference between the centers in unilateral aphakia 16.9% (95% CI, 15.2–18.7) in the south and 14.55 (95% CI, 12.0–17.0) in the north. Of those with unilateral aphakia/pseudophakia, 830 (82%) had an unoperated cataract in the fellow eye, of whom 18% (n = 149) had presenting vision in the better eye <6/60. Women were more likely to have had unilateral or bilateral aphakia/pseudophakia; the age- and center-adjusted ORs were 1.21 (95% CI, 1.03–1.42; *P*<0.02) and 1.30 (95% CI, 1.10–1.55 *P*<0.002), respectively.

Of the 3241 people with unoperated cataract in ≥1 eye, 60% had presenting vision of <6/18 to 3/60 and 12% had vision <3/60. In the operated eye, VA differed by type of operation. Of those with unilateral aphakia (n = 208), 32% had presenting vision of <6/18 to 3/60, and 54% had presenting vision of <3/60. Of those with unilateral pseudophakia (n = 806), the corresponding figures for presenting vision were 46% and 19%, respectively. Of those with bilateral aphakia (n = 271), 39% had presenting vision in the better eye of <6/18 to 3/60 and 26% had presenting vision of <3/60. For bilateral pseudophakia (n = 455), the corresponding figures were 39% and 3%, respectively. For those with aphakia in 1 eye and pseudophakia in the other (n = 120), the figures were 60% and 10%, respectively. Summing these results across all bilateral people who were operated on (n = 846), 42% had VA <6/18 to 3/60 and 11% had VA <3/60.

## Discussion

We found very high prevalence rates of cataract in people aged ≥60 years in centers in both north and south India. We chose to study an older age group than previous studies in India. There has so far been much less information on the older population in India, especially in the oldest age groups. Our study included >2000 people aged ≥70 years compared with <500 in other studies in India.^4,5^ Additionally, an older age group was preferable for the collection of data on age related macular degeneration, the other principal outcome of the INDEYE study.

The areas in our study were chosen to represent the typical population in the catchment area of each center (excluding the city of Delhi, Gurgaon city, and Pondicherry city). Our results therefore do not apply to the city populations served by the participating hospitals where cataract surgery uptake and cataract prevalence may be different. We also cannot assume that our results are generalizable to other populations in the same area. Both the RPC and AEH have an active outreach program, which may lead to a higher cataract surgery uptake than in other areas. Nonetheless we did observe a high proportion of people in both study areas with unoperated cataracts.

The response rates were high (78%). Although nonresponse was higher in the ≥80 age group, there was no response bias in other characteristics such as socioeconomic status or gender. It is possible that the lower response rate in the oldest age group may have led to a biased prevalence estimate, but we have no information to judge the direction of any bias resulting from nonparticipants being more or less likely to have cataract. Comparing results across studies is impeded by differences in methods of measuring and grading cataract, whether the denominator for the prevalence is the study population or only those with gradable lens images, the age and gender distribution of the study population, and the precision of the results. Because very few studies have published 95% CIs, the point estimates do not include the range of possible prevalence. Comparisons by type of opacity are even more problematic because of differences in methods, definitions, and the cataract surgical rate in the surveyed populations because information on the type of cataract before operation is not usually available. Studies describing the prevalence of unoperated cataract vary according to access and uptake of cataract surgery eye care. In our study, we have reported the prevalence of both operated and unoperated cataract as an overall measure of any cataract (past or present). These points need to be borne in mind when discussing results across studies. Our results for the prevalence of all cataracts, including aphakia/pseudophakia, are similar to those reported from 2 previous large studies in India^4,5^ (both in south India; Table 5; available online at http://aaojournal.org). There were some differences in grading between the studies. Although both previous studies used LOCS III grading for nuclear cataract, our definition of nuclear cataract was more stringent. The Aravind Comprehensive Eye Study and Andhra Pradesh Eye Disease Study used a cutpoint on the LOCS III scale of nuclear ≥3, whereas we used a cutpoint of ≥4. Whereas the Aravind Comprehensive Eye Study used identical cutpoints on LOCS III for cortical and PSC as in our study, the Andhra Pradesh Eye Disease Study used the Wilmer grading scheme and cutpoints for cortical and PSC that correspond with LOCS III grade 3 cortical and grade 1 PSC. The estimates for nuclear and PSC from Andhra Pradesh Eye Disease Study are therefore based on a lower threshold than our study or Aravind Comprehensive Eye Study. Our estimates are also slightly lower than from our previous feasibility study.^6^ In that study, we used LOCS II with a threshold of ≥2 for nuclear cataract.

Our prevalence rates of unoperated and operated cataract were similar to comparable age groups in other Asian studies. In the Tanjong Pagar study in Singapore,^9^ the Meiktila Eye Study in Myanmar,^10^ and a study in rural Indonesia,^11^ all using LOCS III and a classification of nuclear cataract ≥4, the prevalence in people aged ≥60 ranged from 72%^10^ to 87%.^9,11^ The slightly lower proportion of cataract in Myanmar could be due to a stricter classification of cortical cataract (≥4), but the number in the older age group was small and the 95% CIs, although not reported, were likely to be wide. The lowest prevalence from the Asian studies was reported from the Shih-Pai study in Taiwan,^12^ even though this study used a lower threshold for nuclear opacity of ≥2. The authors of the Shih-Pai study reported that the study was conducted in a prosperous area of Taipei, and that the nonresponse rate (33%) was higher among older people, women, and those with lower education. The lower prevalence might therefore reflect both bias in the sample and a higher income setting than other studies in India and Asia. In a study pooling the results from several Western populations,^3^ the prevalence of unoperated cataract ranged from 15.5% in the 60- to 64-year-old group to 68% in those aged ≥80 and for any aphakia/pseudophakia from 3% to 29% (Table 5). These results suggest that cataract is more common in Asia, including India, in younger age groups (e.g., 60–64), irrespective of cataract surgery, but by the age of ≥80 years in both Western and Asian populations, the overwhelming majority of people either have a cataract or have been treated for a cataract.

As with other studies in Asia and Western countries, the dominant type of cataract was nuclear (Table 5). The incidence of nuclear opacities seem to be more strongly age related than cortical or PSC opacities.^13–15^ In the Physicians Health study, the age-specific incidence rates of nuclear cataract were approximately double that of cortical or PSC opacity.^15^ Using a cutpoint of LOCS III ≥3, cortical opacities were lower in our study compared with other studies in India and Asia (Table 5). If we used a cutpoint of LOCS III ≥2, our results were closer to other studies (23%). The exception was the Tanjong Pagar study, with very high rates of cortical cataract, LOCS III ≥2 (62%) in those aged 60 to 81 years. Cortical opacities in Western populations are variable; some studies report results broadly comparable with ours,^16,17^ whereas in others the rates seem to be higher.^18,19^ Results in studies of Hispanic Americans^20^ and African Caribbeans,^21^ both based on LOCS II grading definitions of ≥2, produced the highest estimates (28% of those aged 60–69 and 46% of those aged ≥70 in Hispanic Americans and 49% and 72%, respectively, in the Barbados Eye Study). The prevalence of PSC was broadly similar to other studies in India and Asia, although the prevalence rates for the Shih-Pai^12^ and Beijing studies^22^ seemed to be slightly lower. In contrast, the prevalence rates of PSC opacities in Western populations are consistently lower with rates of around 5% to 8% reported for those 60 to 69 years old and around 7% to 14% for those ≥70 years old.^16–21^

Differences between populations in cataract prevalence and especially in the cataract subtypes may reflect environmental or genetic factors. The evidence that ultraviolet radiation is a risk factor for cataract is strongest for cortical cataracts.^23^ Exposure to ultraviolet radiation depends on latitude, occupation, and behavioral factors, but it seems unlikely that the lower prevalence of cortical cataract in India, compared with Hispanic and African Caribbean populations, could be due to lower exposures to ultraviolet radiation. Genetic factors have also been most strongly identified for cortical cataract,^24^ although few genes have been identified. Recently variants in the *EPHA2* gene have been found to be associated with cortical cataracts, and to a lesser extent with nuclear cataracts.^25,26^ The studies, were conducted in individuals of European ancestry and, to date, information is lacking on the association and allele prevalence in groups of other ancestral origins.

We found a higher prevalence of cataract in women compared with men. This was observed for all types of cataract, both unoperated cataracts, and for all operated cataracts. Women were more likely to have undergone cataract surgery compared with men. Many studies worldwide have reported a higher prevalence of cataract among women,^3,5,27^ although in some studies this varied by the type of opacity, being found only for cortical opacities,^4,6,19,22^ or cortical and nuclear,^21^ or nuclear only,^20^ nuclear and PSC,^11^ or all 3 types (cortical, nuclear, and PSC).^12^ Studies examining the incidence of cataract have also reported higher rates among women than men.^13,14^ Lower cataract surgical coverage by women has been documented in many populations^27^ and is a major priority focus for organizations such as Vision 2020 (available: http://www.v2020.org/; accessed January 15, 2010). Our results suggest that the higher rates of cataract in women in our study are not explained solely by differential access to health care, but may be due to other factors such as higher levels of exposures to risk factors such as biomass cooking fuels or intrinsic differences such as hormonal factors.

We observed some differences between the centers in the prevalence of cataract types. Nuclear cataract was higher in north India (48%) compared with south India (38%). For the other types of cataract and for any unoperated cataract, although the differences in the prevalence were significant, the magnitude of the differences was much smaller. The lower prevalence of nuclear cataract in the south might partially be explained by the higher rate of cataract surgery in the south because overall there was no difference between the centers for all unoperated and operated cataract considered together. Other explanations for differences between north and south in the prevalence of type-specific cataracts include environmental, nutritional, and genetic factors. The INDEYE study has collected data on potential risk factors including diet, tobacco use, biomass fuels, and other lifestyle factors. Future analyses will examine the association of these factors with cataract in north and south India. Stored DNA will also facilitate exploration of genetic differences.

## Figures and Tables

**Table 1:** Characteristics by response to lens examination

Characteristics	Non responders⁎ to lens examination† n=1647	Responders to lens⁎ examination† n=5871	p
Study center			
North India	787 (47.8)	2799 (47.7)	0.9
South India	860 (52.2)	3072 (52.3)	
Male	765 (46.4)	2794 (47.6)	0.5
Age groups (y)			
60-64	477 (29.0)	2141 (36.5)	< 0.0001
65-69	386 (23.4)	1592 (27.1)	
70-74	329 (19.9)	1166 (19.9)	
75-79	204 (12.4)	587(10.0)	
80+	251 (15.2)	385 (6.6)	
Land holdings			
No land holdings	1082 (65.7)	3855 (65.7)	0.5
> 0 – <10 acres land	512 (31.1)	1864 (31.8)	
≥ 10 acres land	53 (3.2)	152(2.6)	
Rural area	1178 (71.5.)	4333 (73.8)	0.1
Scheduled caste	352 (21.4)	1116 (19.1)	0.2
Illiterate	1072 (65.1)	3660 (62.3)	0.06

⁎ Responders defined as attended the hospital and underwent a lens examination

† Percentages in parentheses are based on the denominator in the heading of each column

**Table 3:** Age and sex specific prevalence % by type of cataract and for all unoperated and operated cataracts by study center

	North India				South India			
	60-64 n=1029	65-69 n=696	70+ n=1074	60+ n=2799	60-64 n=1112	65-69 n=896	70+ n=1064	60+ n=3072
Any Nuclear⁎								
Men	33.3	45.0	59.8	47.9	26.2	32.4	43.2	34.7
95% CI	28.0-38.5	39.5 -50.4	55.2-64.5	43.9-51.9	20.1-32.4	28.4-36.4	36.8-49.5	30.6-38.8
Women	41.1	51.8	54.6	48.2	35.9	42.4	45.7	40.8
95% CI	37.2-45.0	45.8-57.7	50.0-59.3	45.1-51.3	31.3-40.4	37.1-47.8	40.1-51.4	38.3-43.3
Any Cortical‡								
Men	4.3	7.5	8.3	6.9	5.3	11.2	10.5	9.1
95% CI	2.0-6.5	4.1-11.1	5.7-10.9	5.3-8.5	3.2-7.3	7.9-14.5	8.2-12.8	7.2-10.9
Women	7.7	8.7	8.6	8.3	10.0	11.6	12.6	11.2
95% CI	4.9-10.6	6.0-11.5	5.3-12.0	6.5-10.0	7.3-12.8	8.7-14.4	9.7-15.4	9.7-12.7
Any PSC§								
Men	14.5	19.8	23.8	19.9	15.0	13.6	18.8	16.1
95% CI	11.1-17.9	15.9-23.6	20.6-27.1	17.6-22.2	10.3-19.6	9.6-17.6	16.1-21.5	13.7-18.4
Women	17.8	24.0	26.2	22.1	13.5	20.0	23.7	18.5
95% CI	14.7-20.8	19.3-28.7	22.1-30.2	20.1-24.0	10.0-17.0	16.6-23.3	19.3-23.1	16.3-20.6
Any unoperated cataract∥								
Men	41.6	55.3	68.2	56.7	36.1	48.1	56.8	47.8
95% CI	35.8–47.3	50.3–60.3	64.4–71.9	53.2-60.2	29.5-42.8	42.7-53.5	50.1-63.0	43.5-52.1
Women	48.7	65.7	66.9	58.9	50.9	56.3	66.4	57.2
95% CI	44.5–52.9	60.1-71.3	63.1–70.7	56.2-61.7	46.5–55.3	51.3-61.3	61.2-71.6	54.2-60.1
Any operated or unoperated cataract								
Men	47.5	67.8	90.2	71.4	46.5	68.8	83.3	67.5
95% CI	40.9–54.1	63.0-72.5	88.4–92.0	68.4-74.4	40.3–52.7	63.3-74.4	80.0-86.7	64.6-70.4
Women	56.7	85.8	94.9	76.5	62.8	75.4	91.3	75.1
95% CI	52.8–60.7	81.1-90.0	93.31–96.5	73.7-79.4	57.8–67.7	71.7-79.1	88.8-93.8	72.8-77.5

CI= Confidence Interval

⁎ any nuclear cataract (pure or mixed) defined as Lens Opacity Classification System III (LOCS III)^4^

‡ any cortical cataract (pure or mixed) defined as LOCS III^3^

§ any PSC (posterior subcapsular cataract) (pure or mixed) defined as LOCS III^2^

∥ Any unoperated cataract defined as any nuclear cataract or cortical cataract or PSC cataract or with dense opacities

**Table 5:** Prevalence % of cataract in the India Study of age-related eye disease (INDEYE) and published studies

Study Location Cataract grading	Cataract type	Ages 60-69 N Prevalence (95% CI)		Ages 70+ N Prevalence (95% CI)		
INDEYE		N= 1725		N= 1074		
North India	Nuclear ≥ 4,	42.2 (38.9-45.5)		57.5 (53.3-61.7)		
LOCS III	Cortical ≥3	7.1 (5.6-8.5)		8.5 (6.2-10.8)		
	PSC ≥2	18.7 (16.3-21.0)		24.9 (21.7-27.9)		
	Any untreated cataract	51.8 (48.7 – 54.9)		67.6 (64.6 -70.6)		
	Any cataract	62.7 (59.2 – 66.2)		92.3 (90.9 – 93.6)		
INDEYE		N= 2008		N= 1064		
South India	Nuclear ≥ 4,	34.5 (31.2-37.8)		44.4 (39.6-49.1)		
LOCS III	Cortical ≥3	9.6 (8.3-10.9)		11.5 (9.3-13.3)		
	PSC ≥2	15.4 (13.2 -17.6)		21.1 (18.3-23.8)		
	Any untreated cataract	48.3 (45.0 -51.5)		61.3 (56.7 – 65.8)		
	Any cataract	63.3 (60.2 -66.5)		87.0 (84.5 – 89.5)		
ACES^4^		N=1201		N= 417		
South India	Nuclear ≥ 3	51.5		63.0		
LOCS III	Cortical ≥3	-		-		
	PSC ≥2	-		-		
	Any untreated cataract	81.3		79.4		
	Any cataract	87.8		91.1		
APEDS^5^		N= 899		N= 352		
South India	Nuclear ≥ 3	50.8		67.2		
LOCS III	Cortical Wilmer ≥2	26.8		31.8		
	PSC ≥1	29.9		37.7		
	Any untreated cataract	-		-		
	Any cataract	68.4		86.1		
Tanjong Pagar^9^		N=338		70-81, N=300		
Singapore	Nuclear≥4	57.8		84.7		
LOCS III	Cortical≥2	53.9		72.2		
	PSC≥2	14.5		30.3		
	Any untreated cataract	-		-		
	Any cataract	81.1		94.3		
Shih-Pai^12^		N=481 (65-69)		N=880		
Taiwan	Nuclear≥2	33.3		42.0		
LOCS III	Cortical≥2	19.3		23.3		
	PSC≥2	6.4		10.7		
	Any untreated cataract	41.4		49.1		
	Any cataract	48.0		65.3		
Beijing Study^22^		N=1274		N=486		
China	Nuclear≥3	87.0		95.8		
AREDS^∥^	Cortical≥5%	17.1		33.0		
	PSC ≥1%	5.6		15.6		
Meiktila Eye Study		N=433		N=353		
Myanmar^10^	Nuclear≥4	48.5		73.7		
LOCS III	Cortical≥4	21.9		39.1		
	PSC≥2	9.9		19.3		
	Any untreated cataract	58.4		83.3		
	Any cataract	60.0		86.7		
Pooling project^3^±		60-64	65-69	70-74	75-79	80+
	Any untreated cataract	15.5 (14-17)	25.0 (23-27)	36.9 (35-39)	49.9 (47-53)	68.3 (65-71)
	Aphakia/Pseudophakia	3.0 (3.0-3.3)	5.3 (5-6)	9.1 (8-10)	14.6 (14-16)	29.2 (26-32)

CI= Confidence Interval LOCS= Lens Opacity Classification System ACES= Aravind Comprehensive Eye Survey APEDS= Andhra Pradesh Eye Disease Study AREDS= Age Related Eye Disease Study

± Any untreated cataract= Nuclear cataract Wilmer≥3 or LOCS II≥3 or Wisconsin≥4 or Cortical≥25%of lens or PSC≥1mm

**Table 2:** Age- and Gender-Standardized Prevalence Percent of Cataract by Type and Study Center

Cataract Type	North India (*n* = 2799⁎)		South India (*n* = 3072⁎)		*P*	Both Centers (*n* = 5871⁎)	
	Prevalence (n)	95% CI	Prevalence (n)	95% CI		Prevalence (n)	95% CI
Pure nuclear‡	28.9 (809)	27.2–30.5	24.4 (750)	22.9–25.9	0.01	26.5 (1559)	24.7–28.4
Pure cortical§	2.3 (64)	1.8–2.9	4.3 (134)	3.6–5.0	<0.001	3.4 (198)	2.8–4.0
Pure PSC∥	4.8 (96)	4.0–5.5	3.5 (146)	2.8–4.2	<0.05	4.1 (242)	3.5–4.8
Any pure or mixed nuclear	48.0 (1346)	46.1–49.8	38.0 (1165)	36.6–39.7	<0.0001	42.8 (2511)	40.2–45.3
Any pure or mixed cortical	7.6 (213)	6.6–8.6	10.2 (314)	9.1–11.3	0.004	9.0 (527)	8.1–9.9
Any pure or mixed PSC	21.0 (589)	19.5–22.5	17.4 (533)	16.1–18.8	0.003	19.1 (1122)	17.8–20.3
Any unoperated cataract⁎⁎	57.8 (1620)	56.0–59.6	52.9 (1621)	51.1–54.6	0.01	55.2 (3241)	53.2–57.2
Any operated or unoperated cataract	73.8 (2073)	72.3–75.3	71.8 (2198)	70.3–73.3	0.3	72.7 (4271)	71.1–74.4

CI = confidence interval; PSC = posterior subcapsular cataract.

⁎ Based on all those who attended the hospital clinic examination and underwent clinical examination or lens photography or both.

‡ Nuclear cataract defined as Lens Opacity Classification System III (LOCS III) ≥ 4.

§ Cortical cataract defined as LOCS III ≥ 3.

∥ Posterior subcapsular cataract defined as LOCS III ≥ 2.

⁎⁎ Any unoperated cataract defined as any nuclear cataract or cortical cataract or PSC or with dense opacities.

**Table 4:** Association of Age and Gender with Cataract Type, Unoperated Cataract, and All Cataract

Type of Cataract	Age Groups (y)				Gender		
	60–64	65–69 OR (95% CI)	≥70 OR (95% CI)	*P* Trend	Men	Women OR (95% CI)	*P* OR (95% CI)
Any nuclear‡	1	2.34 (2.01–2.73)	7.34 (5.85–9.21)	<0.0001	1	1.82 (1.59–2.08)	<0.0001
Any cortical§	1	2.65 (2.10–3.35)	7.06 (5.18–9.63)	<0.0001	1	2.07 (1.67–2.56)	<0.0001
Any PSC∥	1	2.41 (1.98–2.92)	7.48 (5.72–9.78)	<0.0001	1	1.80 (1.48–2.18)	<0.0001
Any unoperated cataract⁎⁎	1	2.35 (2.03–2.71)	7.01 (5.65–8.70)	<0.0001	1	1.81 (1.58–2.07)	<0.0001
Any operated or unoperated cataract††	1	2.57 (2.23–2.96)	8.04 (6.48–9.97)	<0.0001	1	1.80 (1.59–2.02)	<0.0001

CI = confidence interval; OR = odds ratios adjusted for age for effects of gender and for gender for effects of age; PSC = posterior subcapsular cataract.

‡ Includes 2511 with any nuclear cataract (pure or mixed nuclear cataracts) defined as Lens Opacity Classification System III (LOCS III) ≥ 4 compared with 1600 people with no cataract of any type or lens opacities or aphakia/pseudophakia.

§ Includes 527 with any cortical cataract (pure or mixed) defined as LOCS III ≥ 3 compared with 1600 people with no cataract of any type or lens opacities or aphakia/pseudophakia.

∥ Includes 1122 with any PSC (pure or mixed posterior subcapsular opacity) defined as LOCS III ≥ 2 compared with 1600 people with no cataract of any type or lens opacities or aphakia/pseudophakia.

⁎⁎ Includes 3241 with any unoperated cataract (any nuclear cataract or cortical cataract or PSC cataract or with dense opacities) compared with 1600 people with no cataract of any type or lens opacities or aphakia/pseudophakia.

†† Includes 4271 with any operated or unoperated cataract compared with 1600 people with no cataract of any type or lens opacities or aphakia/pseudophakia.
